# Transcriptome analysis of secondary cell wall development in *Medicago truncatula*

**DOI:** 10.1186/s12864-015-2330-6

**Published:** 2016-01-05

**Authors:** Huanzhong Wang, Jung Hyun Yang, Fang Chen, Ivone Torres-Jerez, Yuhong Tang, Mingyi Wang, Qian Du, Xiaofei Cheng, Jiangqi Wen, Richard Dixon

**Affiliations:** Department of Plant Science and Landscape Architecture, University of Connecticut, 1390 Storrs Rd., Storrs, CT 06269 USA; Plant Biology Division, Samuel Roberts Noble Foundation, 2510 Sam Noble Parkway, Ardmore, OK 73401 USA; Department of Biological Sciences, University of North Texas, 1155 Union Circle, Denton, TX 76203 USA

**Keywords:** Transcriptome, Expression, Secondary cell wall, Development, Medicago truncatula

## Abstract

**Background:**

Legumes are important to humans by providing food, feed and raw materials for industrial utilizations. Some legumes, such as alfalfa, are potential bioenergy crops due to their high biomass productivity. Global transcriptional profiling has been successfully used to identify genes and regulatory pathways in secondary cell wall thickening in *Arabidopsis*, but such transcriptome data is lacking in legumes.

**Results:**

A systematic microarray assay and high through-put real time PCR analysis of secondary cell wall development were performed along stem maturation in *Medicago truncatula*. More than 11,000 genes were differentially expressed during stem maturation, and were categorized into 10 expression clusters. Among these, 279 transcription factor genes were correlated with lignin/cellulose biosynthesis, therefore representing putative regulators of secondary wall development. The b-ZIP, NAC, WRKY, C2H2 zinc finger (ZF), homeobox, and HSF gene families were over-represented. Gene co-expression network analysis was employed to identify transcription factors that may regulate the biosynthesis of lignin, cellulose and hemicellulose. As a complementary approach to microarray, real-time PCR analysis was used to characterize the expression of 1,045 transcription factors in the stem samples, and 64 of these were upregulated more than 5-fold during stem maturation. Reverse genetics characterization of a cellulose synthase gene in cluster 10 confirmed its function in xylem development.

**Conclusions:**

This study provides a useful transcriptome and expression resource for understanding cell wall development, which is pivotal to enhance biomass production in legumes.

**Electronic supplementary material:**

The online version of this article (doi:10.1186/s12864-015-2330-6) contains supplementary material, which is available to authorized users.

## Background

Legumes include many agriculture important grain, pasture and agroforestry species that are well known for their ability to fix nitrogen through root nodules [1, 2]. Alfalfa (*Medicago sativa*) is a major forage legume, with an average annual value of more than $8 billion in the USA alone [3]. It’s high biomass productivity also makes alfalfa a potential bioenergy legume [4]. Most cultivated legumes, including alfalfa, are multiploid with complex segregation and inheritance patterns [2, 5]. Barrel medic (*Medicago truncatula*) is diploid with a relatively small genome, and has been adopted as a model species for legume genomics [6–8]. In addition, *M. truncatula* is an important self-regenerating annual pasture species, especially in southern Australia [9]. *M. truncatula* is also grown in rotation with cereal crops for improving soil quality [10].

Modulation of secondary cell wall composition, such as reducing lignin content, improves alfalfa quality with better digestibility and higher fermentable sugar yields for biofuel production [11–13], and reduced lignin transgenic alfalfa with increased digestibility has recently been de-regulated in the US by USDA-APHIS [14] and is in commercial production. The bioengineering applications in alfalfa have largely relied on the identification of secondary wall biosynthetic and/or regulatory genes from the model legume *M. truncatula* [12]. Secondary cell wall development in vascular and interfascicular tissues involves a large number of biosynthetic genes and is regulated at the transcriptional level [15, 16]. The NAM, ATAF1/2, and CUC2 (NAC) domain and MYB domain transcription factors (TFs) function as master regulators. The NAC domain TFs include VASCULAR-RELATED NAC-DOMAIN6 (VND6), VND7, NAC SECONDARY WALL THICKENING PROMOTING (NST1), NST2 and SECONDARY WALL-ASSOCIATED NAC DOMAIN 1 (SND1) [17–19]. MYB domain TFs, i.e. MYB46 and MYB83, also function as master regulators for secondary cell wall development, but are downstream of the NAC domain TFs [20, 21]. Many other TFs are further downstream of the NAC and MYB domain master regulators, and form the hierarchical and non-hierarchical regulation networks. The regulatory pathways orchestrate the biosynthesis of cellulose, hemicelluloses and lignin [22]. In *M. tuncatula*, a NAC domain TF was identified as an important regulator of secondary cell wall development, but with distinct regulatory mechanism [23, 24].

Global transcriptional profiling has been successfully used to identify genes and regulatory pathways in secondary cell wall biosynthesis in *Arabidopsis thaliana*, but such transcriptome data and systems analysis of TF functions in stem maturation is still lacking in legumes. The composition of secondary cell walls significantly affects the quality of forage legumes [12, 13, 25]. In addition, cell wall biosynthesis is regulated differently in legumes compared to Arabidopsis [24, 26]. The Affymetrix Medicago genome array has been a critical tool for functional analysis and gene expression studies in legumes [7, 27, 28]. To better understand secondary cell wall development and its regulation in *M. truncatula*, we have performed transcriptome microarray assay and high through-put quantitative real time PCR analysis. A large of number of genes were differentially expressed during stem maturation, and were placed into 10 expression clusters. TFs putatively functioning in secondary wall development were identified based on their expression patterns. Gene co-expression network analysis was used to identify TFs that may regulate the biosynthesis of individual components of the secondary cell wall. We further performed real-time PCR analysis to characterize the expression of 1,045 TFs, and 64 of these were upregulated more than 5-fold during stem maturation. This research provide a useful resource for molecular characterization of secondary cell wall development in legumes.

## Results

### Analysis of secondary cell wall development in the *Medicago* stem

**Fig. 1:**
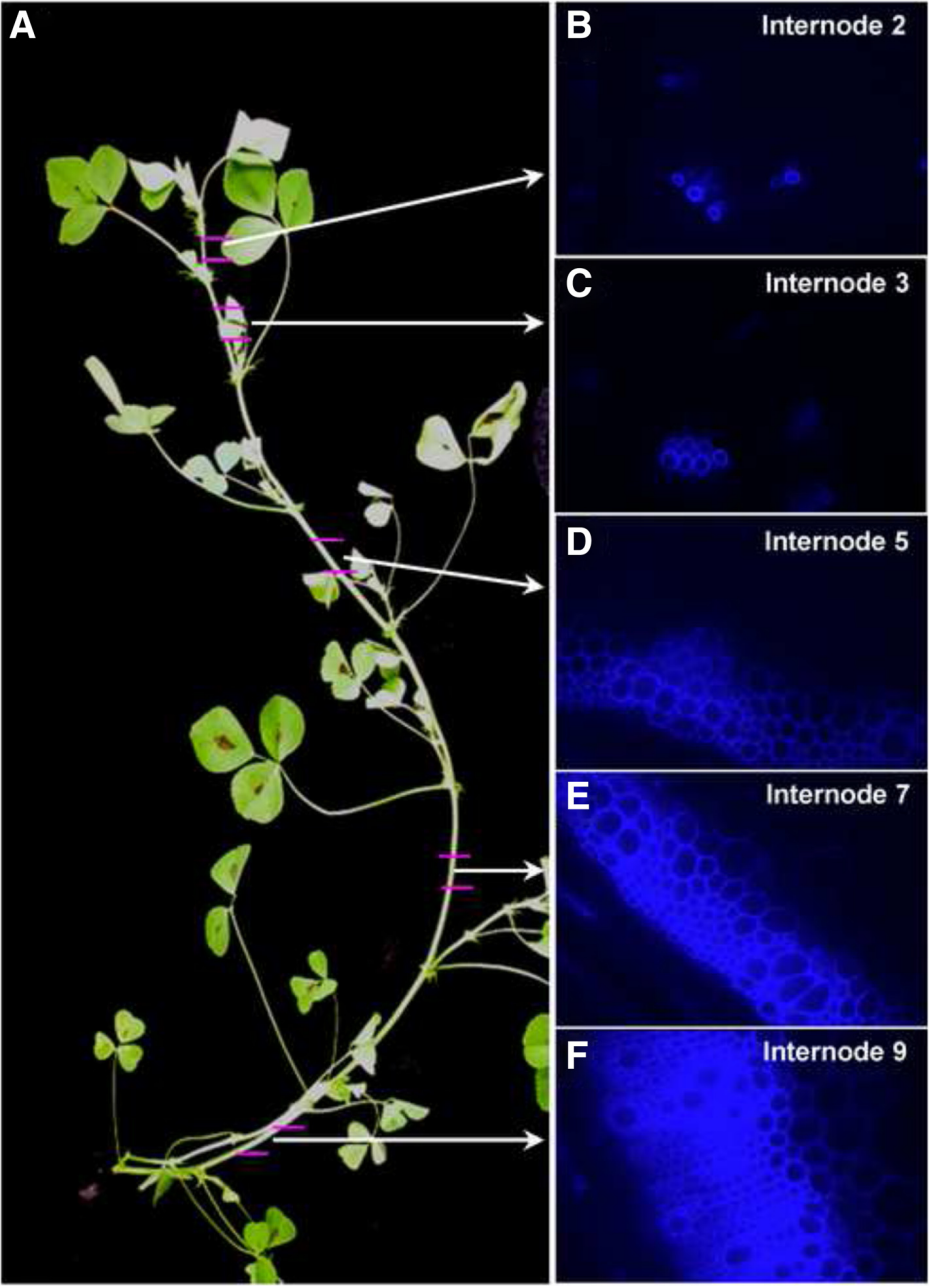
Secondary cell wall development is correlated with stem maturation. **a** A representative stem of 7-week old *M. truncatula* plants. Medicago plants normally have ten to eleven internodes at this stage. **b** to **f** Stem cross sections observed under UV light. The blue color is autofluorescence from lignified secondary cell walls. From the plant apex to the bottom, the internodes become more mature and the stems accumulate more secondary cell walls

To understand the secondary cell wall development in vascular bundles and interfascicular fibers in *M. truncatula*, we characterized the developing primary stems by UV microscopy of cross sections. Stems of 7-week old *Medicago* plants develop 10-11 internodes under greenhouse conditions. Internodes at the middle of the stem grow much longer than the young internodes at the top, or the old internodes at the bottom. In order to collect representative and consistent samples, we chose only the center portion of each internode for histological analysis (Fig. 1a). In internode 2, which is located just below the growing apex, a few primary vascular vessels were observed and showed weak blue autofluorescence due to lignin deposition (Fig. 1b). In the third internode, more vessel elements developed in the vascular region, but no interfascicular fibers were observed (Fig. 1c). In internode 5, vascular bundles were well developed. Interfascicular fibers accumulated a considerable amount of secondary cell wall material, although the autofluorescence signal was still weak compared to the vascular bundle regions (Fig. 1d). In internode 7, both vascular bundles and interfascicular fibers accumulated large amounts of secondary wall material (Fig. 1e). In the mature internode 9, both vascular and interfascicular regions expanded in width, and secondary cell wall development was almost complete (Fig. 1f). Similar to stem development in *Arabidopsis* [29], our histological analysis indicated that the most prominent developmental change during Medicago stem maturation was secondary cell wall differentiation and accumulation of lignocellulosic compounds. The stem maturation analysis in this research is consistent with the results of previous cell wall composition and digestibility assays in Medicago stems [30].

### Microarray analysis of secondary wall development and stem maturation

To characterize the transcriptome profile during *Medicago* stem maturation, we collected stem samples for RNA extraction and subsequent microarray expression analyses. Five internodes, i.e. the aforementioned internodes 2, 3, 5, 7, and 9 from the *Medicago* primary stem, were collected in three biological replicates. Each sample was a pool of 10 segments harvested from the central 2 cm of each internode. These samples represented the different secondary cell wall developmental stages along the stem maturation process (Fig. 1).

The Affymetrix Medicago Genechip genome array contains 61,281 probe sets, most of which (about 50,900) are from *M. truncatula* gene sequences. Genechip analysis has been instrumental in identifying biologically meaningful genes and characterizing gene expression patterns in *M. truncatula* and *M. sativa* [7, 27]. In this study, we used 15 arrays to investigate the transcriptome change during Medicago stem maturation. RNA samples from internode 2 were used as the reference for the remaining internode samples. Genes with expression levels significantly changed between the control (Internode 2) and the other four older internodes were identified using associative analysis [31]. Analysis of the microarray results indicated that 11,380 genes were significantly differentially expressed (*p* < 8.16 × 10^-7^ and fold change ≥ 2) in the relatively more mature internodes. The differentially expressed genes are listed in supplemental data (Additional file 1).

**Fig. 2:**
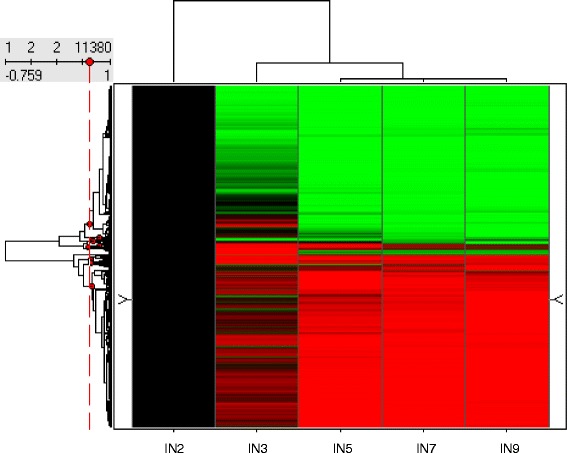
Gene cluster analysis based on expression patterns. Among the 61,281 probe sets in the Affymetrix *Medicago* genome array, 11,380 probe sets showed increased or decreased expression (≥2-fold) in older internodes compared to the second internode. Genes with changed expression were clustered into 10 different categories based on their expression pattern. Red dots indicate the branch point of each cluster. Black indicates no change; green indicates down-regulation and red indicates up-regulation of expression. IN refers to internode number

**Fig. 3:**
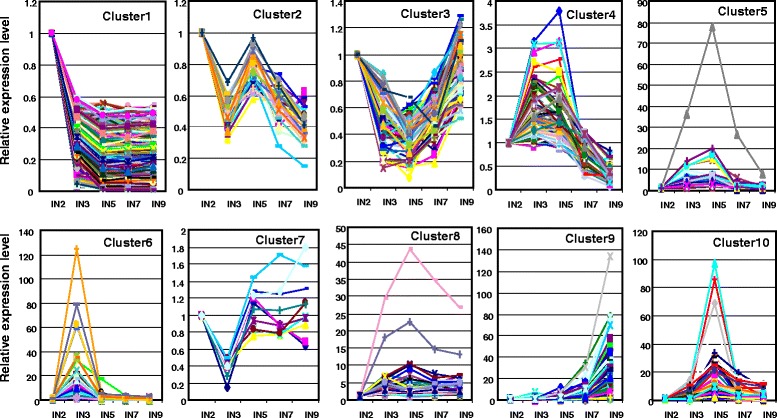
Expression patterns of the 10 gene clusters. Genes in the 10 clusters showed distinct expression patterns in the five characterized internodes. The horizontal axes represent the relative gene expression in internode 2 (IN2), internode 3 (IN3), internode 5 (IN5), internode 7 (IN7) and internode 9 (IN9), respectively

The large number of differentially expressed genes indicates that stem development and secondary cell wall biosynthesis are complex processes that involve many biosynthetic pathways. It is also possible that many of these genes may not be directly involved in cell wall development. To identify which of these genes are important for secondary cell wall development, we performed hierarchical clustering analysis. The differentially expressed genes were placed into 10 clusters based on their expression patterns in the five investigated internodes (Fig. 2 and Additional file 1: Table S1). Interestingly, most of these genes were categorized in either cluster 1 or cluster 10 (Additional file 1: Table S1). In cluster 10, gene expression increased and reached highest expression level in internode 5, and then decreased to a moderate level in the two order internodes (Fig. 3). As shown in Fig. 1, cells in the vascular bundle and interfascicular regions started actively accumulating secondary wall materials in internode 5, indicating that the expression of cluster 10 genes coincides with secondary wall accumulation. We reasoned that genes in cluster 10 are likely have a role in secondary cell wall development, which is supported by analyzing the expression of lignin biosynthetic genes (this research). Genes in clusters 5 and 8 also showed highest expression in internode 5, while their expression pattern slightly different from cluster 10. Genes in these two clusters already showed increased expression in the 3rd internode, in which secondary cell wall biosynthesis is not very active. Despite the noticeable difference in expression pattern, we can’t rule out the possibility that genes in cluster 5 and 8 may also have functions in secondary cell wall development. We therefore include genes from clusters 5, 8 and 10 in our further analysis.

In cluster 1, genes had highest expression in internode 2, and the expression levels progressively decreased along the stem maturation gradient until the fifth internode, and kept at certain levels in the even older internodes (Fig. 3). In contrast, expression of the genes in clusters 2, 3, 4 6 fluctuated along the stem maturation gradient without obvious correlation with secondary wall accumulation. Genes in cluster 7 are down regulated in the 3rd internode, which contradicts with the microscopic observation on secondary cell wall accumulation. Therefore, genes in this cluster are not likely function in secondary cell wall biosynthesis. The expression of genes in cluster 9 increase along stem maturation gradient, and reach the highest level until the oldest 9th internode, in which secondary wall accumulation should have almost completed. We therefore hypothesize that the functions of genes in cluster 9 may not be in secondary cell wall biosynthesis, but rather in the senescence process. Genes in these seven clusters were therefore excluded from further analysis.

### Expression of secondary wall related genes during stem maturation

**Fig. 4:**
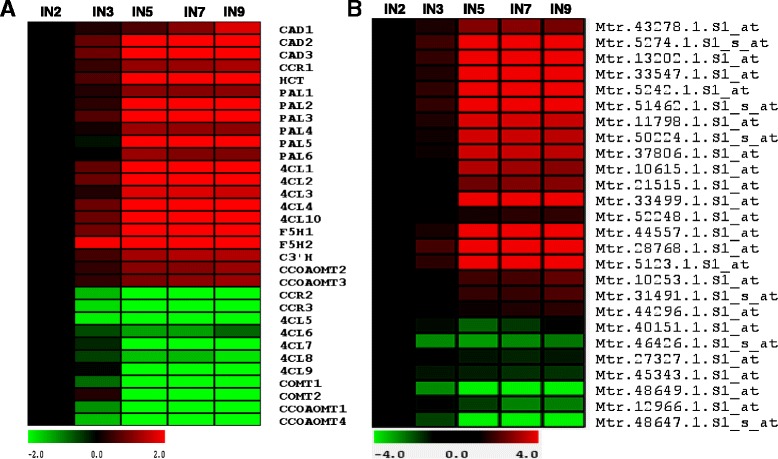
Expression of genes with putative functions in secondary cell wall synthesis. Most of the lignin biosynthetic genes (**a**) and cellulose synthase and cellulose synthase-like genes (**b**) are assigned in cluster 10 (red), but some lignin and cellulose synthetic genes are assigned in cluster 1 (green). Cinnamyl alcohol dehydrogenase (CAD); cinnamoyl CoA reductase (CCR); hydroxycinnamoyl CoA shikimate:quinate hydroxycinnamoyl transferase (HCT); L-phenylalanine ammonia-lyase (PAL); 4-coumaroyl CoA ligase (4CL); ferulic acid/ coniferaldehyde/ coniferyl alcohol 5-hydroxylase (F5H); coumaroylshikimate 3′-hydroxylase (C3′H); caffeoyl CoA 3-*O*-methyl transferase (CCOAOMT); caffeic acid/5-hydroxyferulic acid 3-*O*-methyltransferase (COMT)

Cellulose and lignin are two of the major components of the secondary cell wall. We reasoned that genes responsible for the biosynthesis of cellulose and lignin should be correlated with the stem maturation process. Genes responsible for monolignol biosynthesis have been identified using a comparative genomic approach in *M. truncatula* [12]. Probe sets corresponding to these monolignol synthetic genes were identified through sequence analysis (Additional file 1: Table S2). Expression patterns of these genes were extracted from our transcriptome data and are presented in Fig. 4a. Hierarchical clustering analysis indicated that most of the monolignol biosynthesis genes were upregulated along the stem maturation gradient and were categorized in cluster 10 (red colored, Fig. 4a), but some members were down-regulated and clustered in cluster 1 (green colored, Fig. 4a). This indicates that the annotated monolignol biosynthetic genes in cluster 1 may not be responsible for secondary cell wall biosynthesis during normal plant development. We also analyzed the expression of genes responsible for cellulose biosynthesis. Twenty six probe sets were identified as either putative cellulose synthase genes or cellulose synthase-like genes. Similar to the monolignol biosynthesis genes, these probe sets were categorized either in cluster 1 or in cluster 10 (Fig. 4b). Cellulose synthase genes in cluster 10 are likely to function in secondary wall biosynthesis (see below), while the functions of the putative cellulose synthases categorized in cluster 1 are still unclear.

### Transcription factors correlated with secondary cell wall development

Genes responsible for secondary cell wall development are well known to be under transcriptional regulation in *Arabidopsis*. A large number of transcription factors (TFs) play important roles in regulating secondary wall biosynthetic genes [18, 21, 22, 32–34]. In *M. truncatula*, we previously reported the identification of two secondary cell wall related TFs in the NAC domain and WRKY domain gene families [24, 35, 36], but the regulatory pathways in secondary wall biosynthesis are still largely unknown in *M. truncatula*. To identify novel regulatory genes in secondary cell wall development, we focused our transcriptome data analysis on TFs. In cluster 10 alone, there are 279 TFs that are correlated with secondary cell wall development. There are 8 and 4 TFs in cluster 5 and cluster 8, respectively. Among these 291 transcription factors, the b-ZIP, NAC, WRKY, C2H2 ZF, homeobox, and HSF gene families were over-represented as determined by hypergeometric probability analysis (Table 1).

**Table 1:** Categorization of differentially expressed transcription factors in cluster 5, cluster 8 and cluster 10

Category	Occurrence	*P*-value*
MYB	30	0.921
b-ZIP	28	0.014
NAC	24	0.004
WRKY	24	0.06
C2H2 (ZF)	21	0.115
AP2/EREBP	20	0.687
HB	19	0.122
bHLH	17	0.869
C2C2 (ZF)	8	0.97
HSF	8	0.13
C3H (ZF)	6	0.901
MADS	6	0.949
AUX/IAA	5	0.186
CCAAT-binding	5	0.727
Scarecrow	5	-
RING (ZF)	4	-
ARF	3	0.994
SPF1	3	-
AOPBP	2	-
E2F	2	0.194
GARP	2	0.989
SREBP	2	-
BES1/BZR1	1	0.374
DELLA	1	-
DnaJ protein	1	-
EIL protein	1	0.69
GT-1	1	-
LIM	1	0.374
LOB	1	-
Phaseolin G-box binding	1	-
PHD (ZF)	1	0.999
TCP	1	-
TFIIIB	1	-
TGACG-BP	1	-
Trihelix	1	0.209
Others	34	

*A total of 291 putative transcription factor genes from cluster 5, 8, and 10 were categorized based on functional domains. The distribution as frequencies of occurrence was presented in column 2. *P values were calculated in hypergeometric distribution analysis as the following: The proportion of transcription factors of a given class was compared with the proportion of that class in the whole set of transcription factors. AOPBP, ascorbate oxidase promoter-binding protein. SREBP, sucrose-responsive element binding factor. The classification of the transcription factors can be found at http://mtgea.noble.org/

**Fig. 5:**
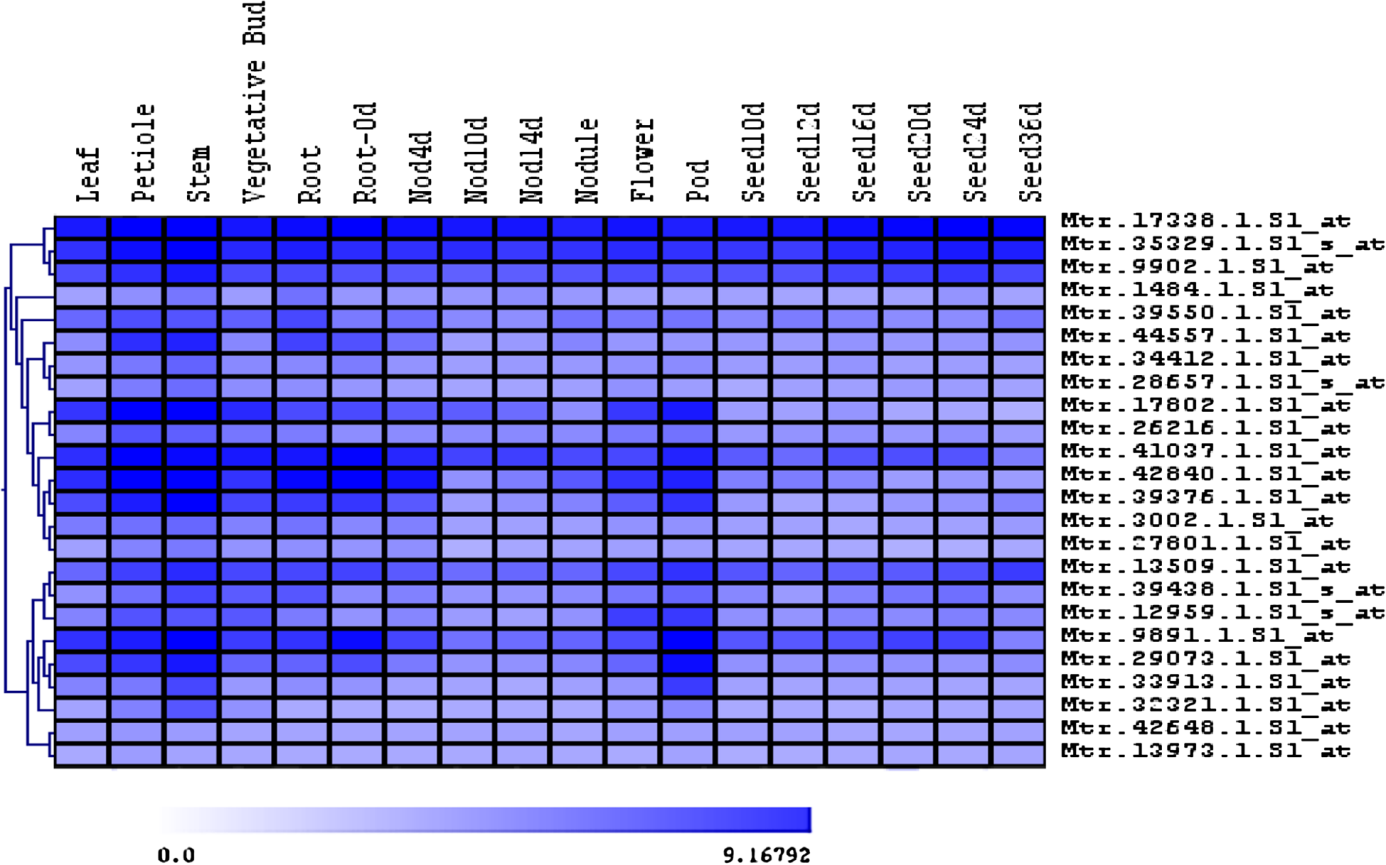
Expression pattern analysis of transcription factor genes highly expressed in secondary wall thickening. Expression of all selected 279 transcription factors was analyzed based on expression patterns in different tissues. Transcription factors highly expressed in tissues with secondary wall thickening was presented in this figure

The large number of TFs (279, *P* = 8.81 × 10^-42^) in cluster 10 may reflect the fact that many coordinated biological pathways are involved in secondary wall development. To understand which TFs are specific to secondary wall biosynthesis, we further analyzed the expression patterns of these genes using the available microarray data from the Medicago Gene Expression Atlas [27]. We reasoned that specific or enhanced expression of TFs in secondary wall bearing tissues would be a strong indication for involvement in secondary wall biosynthesis. We collected expression data of the TFs in 18 different tissues from the gene expression Atlas [27]. Genes with similar expression patterns are clustered together using hierarchical clustering analysis (Additional file 1: Figure S1). TFs in one of the sub-clades are highlighted with a red bar (Additional file 1: Figure S1). These genes are highly expressed in petiole, stem, root and pod, tissues which accumulate secondary cell walls (Fig. 5). This sub-clade included the NAC domain transcription factor *MtNST1* that functions as a master regulator of secondary cell wall development [23, 24]. Other TFs clustering in the same clade may also participate in secondary wall development, and serve as good candidates for reverse genetic analysis.

**Fig. 6:**
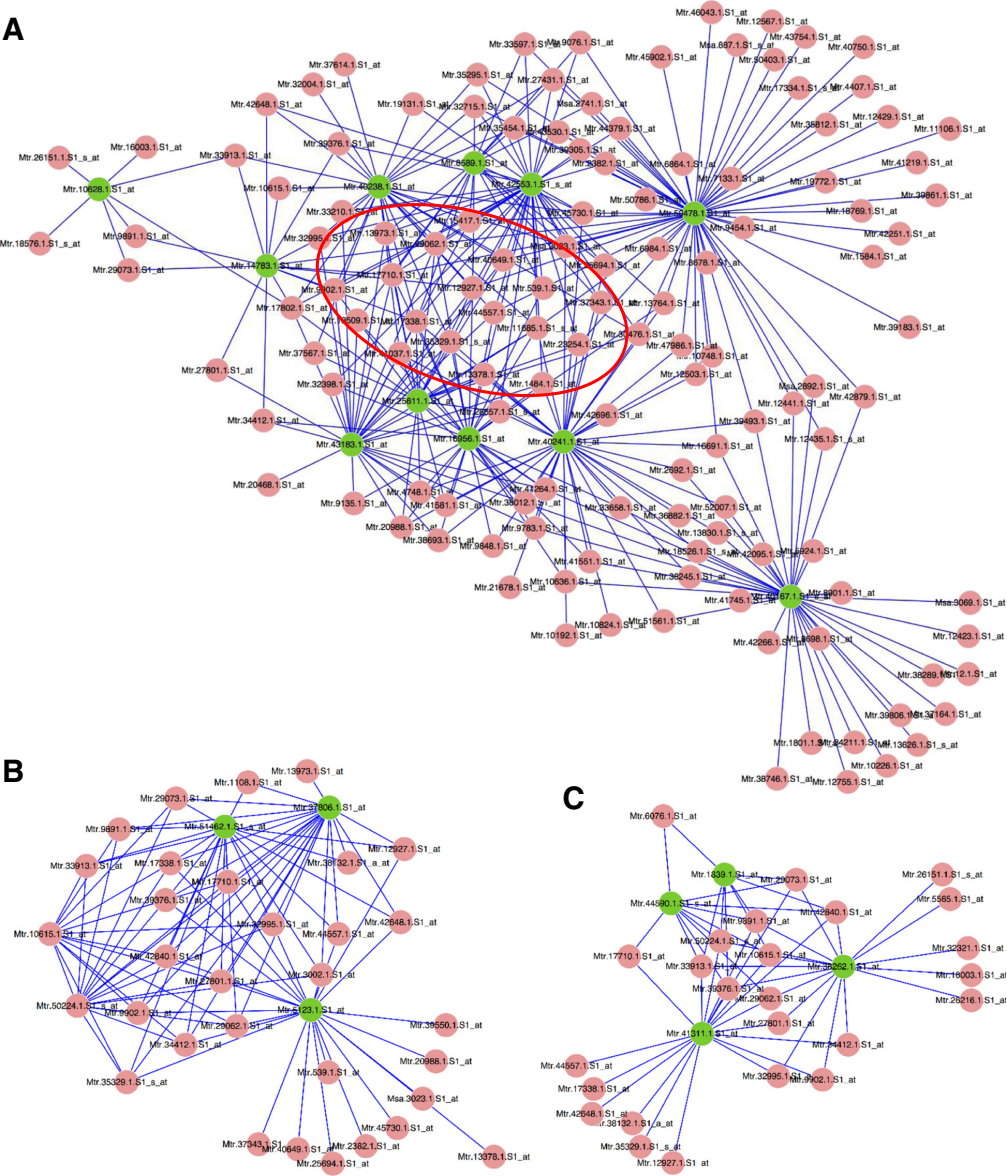
Co-expression network analysis of transcription factors with secondary wall biosynthetic genes. **a**-**c** Networks showing transcription factors co-expressed with 11 lignin biosynthetic genes (**a**), three cellulose synthase genes (**b**), and four hemi-cellulose synthase genes. Transcription factor genes are in brown color and wall biosynthetic genes are in green color. Red oval circles transcription factors connected with four or more lignin synthetic genes. Nodes represent genes, and edges represent significant positive co-expression relationships between genes

Gene co-expression network analysis has been a powerful tool for addressing gene function, and we therefore applied this approach to identify TFs that may regulate the biosynthesis of individual secondary cell wall components. Expression patterns of all putative TFs and genes responsible for lignin, cellulose and hemicellulose synthesis were collected from the Medicago Gene Expression Atlas [27]. Data for leaf, petiole, stem, shoot (split-root experiment), sufficient N_2_, flower, pod, root and split root (nodulating) sufficient N_2_ and the present stem experiment (37 chips in total) were used. In this analysis, only probe sets corresponding to Medicago genes were utilized, while those labeled as “AFFX”, “Sme” or “RPTR” were removed. The resulting 12,576 probe sets were used to calculate the Pearson correlation coefficient values (PCC). TFs connected (|PCC| > 0.8) with lignin, cellulose and hemicellulose synthesis genes are presented in Fig. 6 We included 11 monolignol synthetic genes in this analysis. A large number of the TFs are connected with monolignol biosynthesis, but most of them only connected with 1 or two of the lignin genes. TFs that are highlighted in the red oval are connected to as many as 5 monolignol synthetic genes. These TFs are likely important regulators of the whole monolignol synthetic pathway (Fig. 6a). In contrast, fewer TFs were connected to secondary wall related cellulose and hemicellulose synthesis genes (Fig. 6b and c).

### High through-put quantitative real-time PCR analyses

The Affymetrix Genechip array includes a large portion of the transcriptome from *M. truncatula*, but some genes are missing from the Genechip. Furthermore, the differentially expressed transcripts identified from microarray analysis were not absolutely quantified and need to be confirmed through independent analysis. As a complementary approach to the microarray analysis, we investigated the expression of TFs along stem maturation using large scale real-time PCR. A community resource has been previously established to study transcription factors in *M. truncatula* [37]. This community resource included gene specific oligonucleotide primers for 1045 TFs. Among these, 916 TFs were expressed in the internode samples. The expression of 64 TFs was increased more than 5-fold along the stem maturation gradient (Additional file 1: Table S3). Twenty of them were already detected in the microarray experiment, therefore the expression of these TFs validated the microarray data. Interestingly, 18 of the TFs were present on the Genechip, but their expression changed less than 2-fold in the microarray data, indicating that real-time PCR is the more sensitive analytical method. Furthermore, there were 26 significantly differentially expressed TFs that were not present on the Genechip. Hypergeometric probability analysis indicated that C2H2 Zinc-finger and MYB domain transcription factors are over represented among the 64 TFs (Table 2).

**Table 2:** Distribution of transcription factors with increased expression along stem maturation gradient as determined by high-throughput q-RT PCR

Category	Frequencies of occurrence	*P-value
MYB	10	0.276
AP2/EREBP	5	0.52
C2H2 (ZF)	5	0.285
CCHC (ZF)	4	-
bHLH	3	0.853
b-ZIP	3	0.803
HB	3	0.573
NAC	3	0.625
WRKY	3	0.763
B-box (ZF)	2	-
GRAS	2	0.691
MADS	2	0.647
RING (ZF)	2	-
C2C2 (ZF)	1	0.952
CCAAT	1	0.735
DHHC (ZF)	1	-
Dof (ZF)	1	-
GRF (ZF)	1	-
LOB	1	-
PHD (ZF)	1	-
SBP	1	-
Others	9	

*P-value was calculated similar to Table 1

### Reverse genetics characterization of the *IRX1* gene in *M. truncatula*

Most genes in the lignin biosynthetic pathway have been identified from *Medicago*, and several have been used to improve digestibility and sugar yield in alfalfa [11–13]. Much less is known about the functions of the cellulose synthase genes in legumes. Our microarray analysis indicated that 19 CesA probe sets were categorized in cluster 10 (Fig. 4b red colored probe sets), and may therefore represent bona fide secondary cell wall specific cellulose synthase genes. In *Arabidopsis*, three cellulose synthases form a complex to catalyze cellulose synthesis in the secondary cell well; these are AtCesA4/IRX5, AtCesA7/IRX3/FRA5 and AtCesA8/IRX1/ FRA6 [38–41]. Using blast analysis, we found that one of the probe sets in cluster 10, Mtr.5123.1.S1_at, has highest identity with AtIRX1/AtCesA8. We named the corresponding *Medicago* homolog gene *MtIRX1*. To investigate the biological function of *MtIRX1*, we performed a reverse genetic analysis. Using *MtIRX1* gene-specific primers, three mutant lines were recovered from the Medicago *Tnt1* insertional mutant population. In a previous mutant screening experiment, we had already identified a *Tnt1* insertion in the *MtIRX1* gene and named the mutant *mtirx1*-*1* (NF3892). We therefore renamed the newly identified mutants NF7461, NF8667 and NF17907 as *mtirx1*-*2*, *mtirx1*-*3 and mtirx1*-*4*, respectively. These four mutant lines showed similar irregular xylem phenotypes, and the phenotypes of *mtirx1*-*1* were examined in detail.

**Fig. 7:**
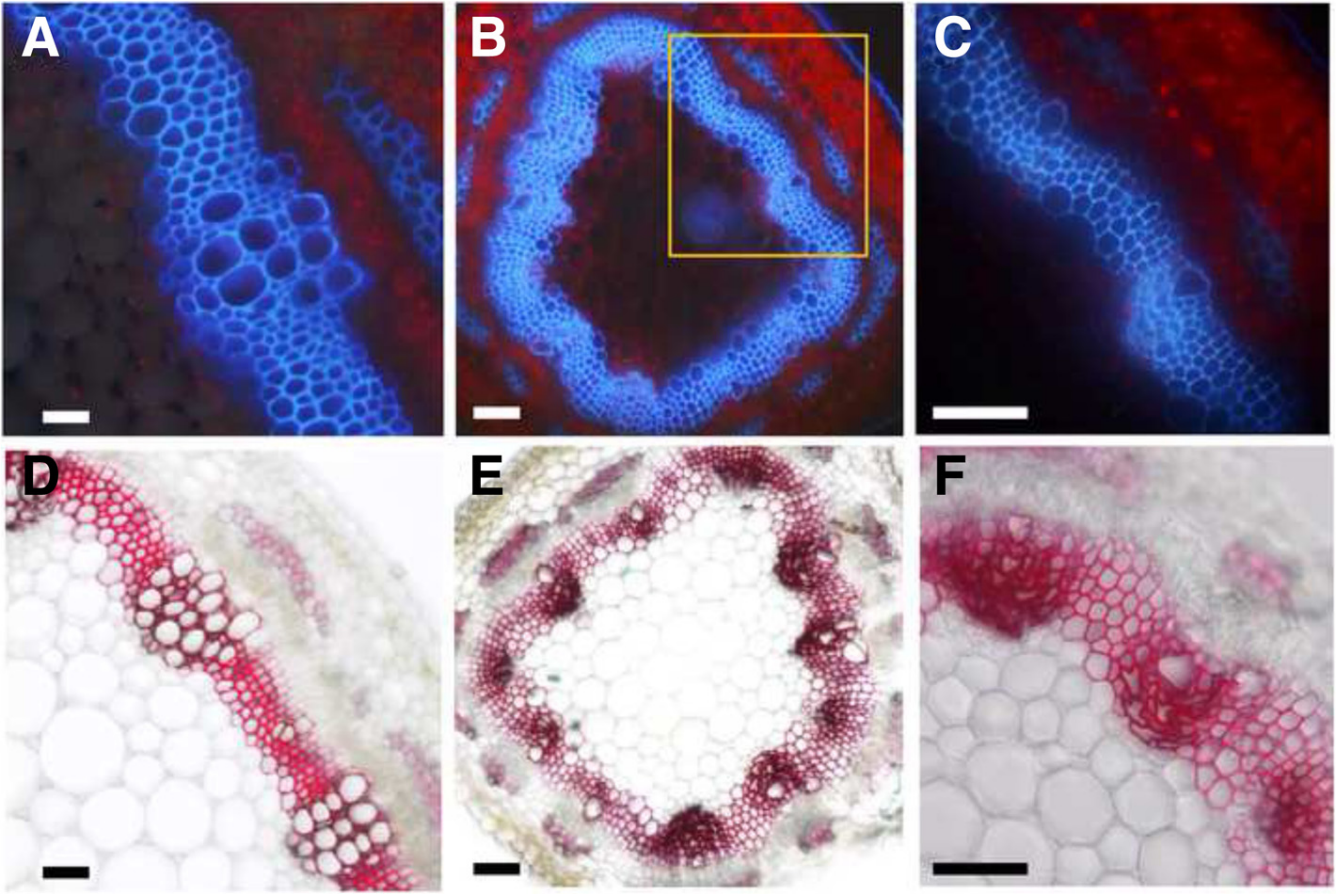
Characterization of the *mtirx1*-*1* mutant by UV microscopy and phloroglucinol staining. **a** to **c**, stem cross sections of wild type (**a**), and the *mtirx1* mutant (**b**) were observed under UV light. **c** A higher magnification of the rectangle marked area in (**b**). Collapsed xylem phenotype is evident. The blue color is the auto-fluorescence of lignin under UV light, and the red color is the auto-fluorescence of chloroplasts. **d** to **f**, cross sections of wild type (**d**) and the *mtirx1* mutant (**e**) after phloroglucinol staining. **f**, Staining of the *mtirx1* mutant at a higher magnification. Bars = 10 μm

In wild type plants, cross sections of the sixth internodes showed well organized and regular shaped vascular vessel and fiber cells (Fig. 7a), while most of the vessel cells were collapsed in the *mtirx1*-*1* mutant plants (Fig. 7b and c). Furthermore, the stems of the mutant plants were thinner than the wild type (Fig. 7a to c). To better characterize the mutant phenotype, we examined the stem cross sections with phloroglucinol staining. Phloroglucinol stains the lignin a bright red color, therefore enabling the visualization of cells with secondary cell walls. Compared to the wild type (Fig. 7d), the xylem vessel cells were totally distorted and collapsed in the *mtirx1*-*1* plants (Fig. 7e and f). Furthermore, the cell walls of the vascular and interfascicular fiber cells in *mtirx1*-*1* plants were visually thinner compared to the wild type plants (Fig. 7b and e).

**Fig. 8:**
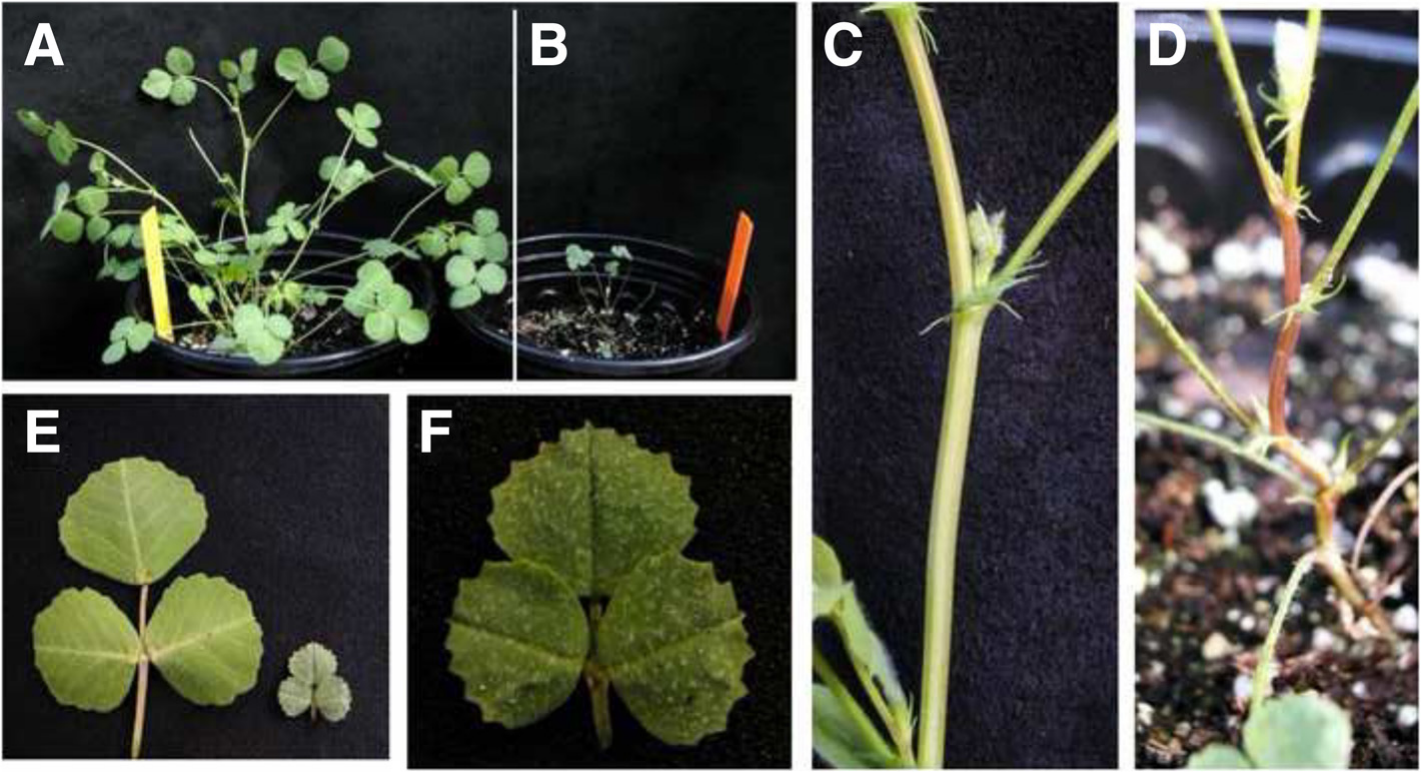
Growth phenotype of *mtirx1* mutant plants. **a** A wild type *M. truncatula* plant. **b**, A representative *mtirx1*-*1* mutant, which is extremely small compared to the wild type. **c** and **d**, Representative stems of wild type (**c**) and the mutant (**d**). **e** Leaves of wild type (left) and mutant (right). **f** The mutant leaf at a higher magnification

In addition to the collapsed xylem, the *mtirx1*-*1* mutant plants also showed obvious growth phenotypes. As shown in Fig. 8, the *mtirx1*-*1* mutants are extremely small (Fig. 8a and b). The mutant stems are much thinner, with excessive pigment accumulation in the epidermal cells (Fig. 8c and d). The mutant leaves are also significantly smaller than the wild type. At higher magnification, the mutant leaves showed a dark green color with evident trichomes covering on the leaf surface (Fig. 8e and f). In *Arabidopsis*, mutations of secondary wall related cellulose synthase genes showed similar irregular xylem phenotypes, as well as retarded plant growth [38–41].

**Fig. 9:**
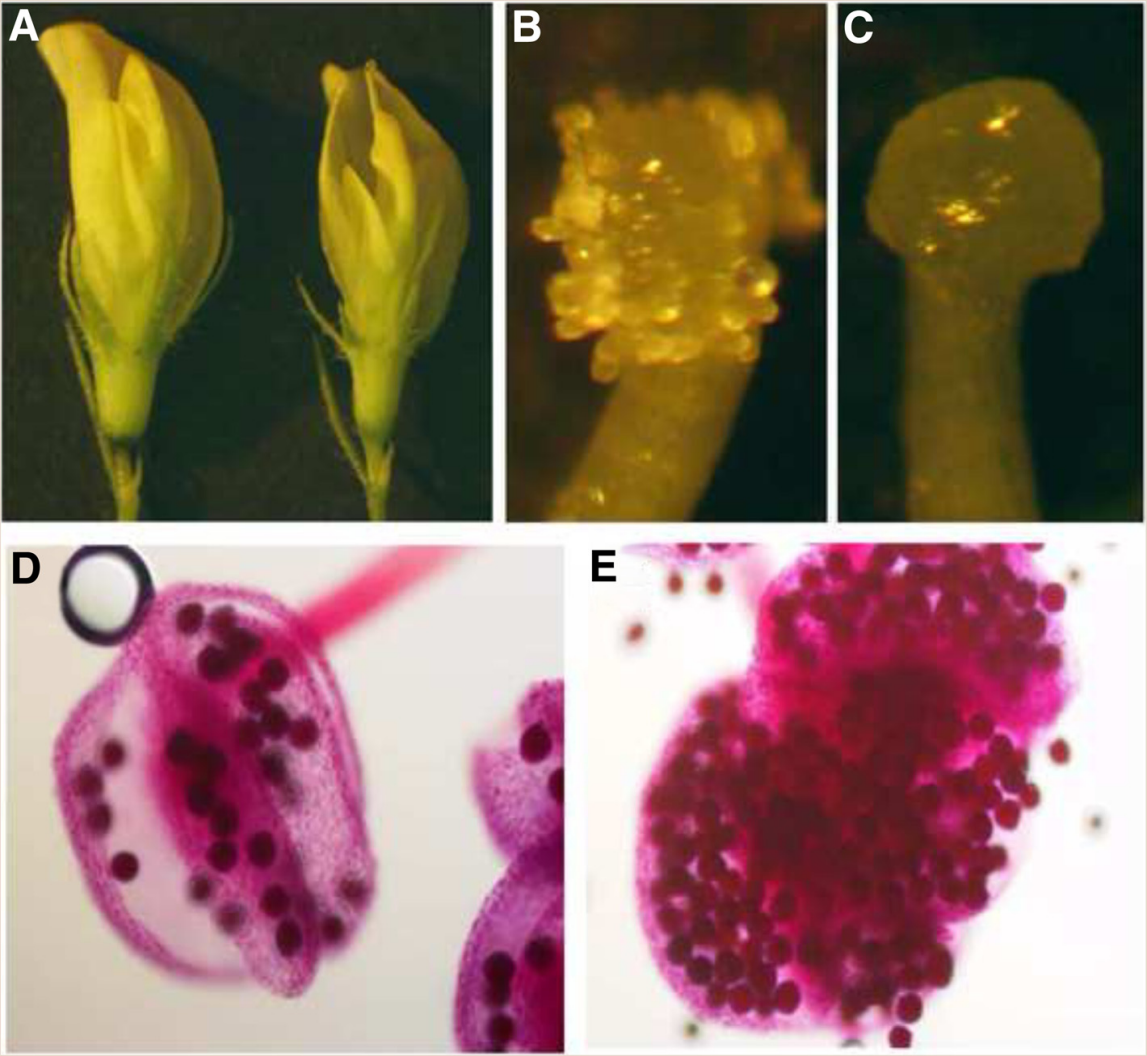
Defects of the reproductive organs in *mtirx1*-*1* mutant plants. **a**, Flowers of the wild type (left) and *mtirx1*-*1* mutant (right), showing the slightly smaller flower of the mutant plants. **b** and **c**, Close-up of the stigmas of wild type (**b**) and *mtirx1*-*1* mutants (**c**) showing the smooth surface of mutant stigma with no pollen attaching on the surface. **d** and **e**, Alexander’s staining showing that pollen is viable in both wild type (**d**) and mutant plants (**e**)

The *mtirx1*-*1* mutant plants could not set seeds under our glasshouse conditions. To investigate what caused the infertile phenotype, we characterized the flower structure in detail. As shown in Fig. 9a, the flowers from the mutant plants are slightly smaller than the wild type; and the stigma of the mutant sticks out of the flower. Unlike the wild type (Fig. 9b), the mutant stigma has a smooth surface with no pollen attached (Fig. 9c). We further characterized the viability of pollen grains using Alexander’s staining. The results indicated that the mutant pollen was as viable as that of the wild type (Fig. 9d and e). Therefore, the infertile phenotype of the *mtirx1*-*1* mutant may be due to defective anther dehiscence. In both *Arabidopsis* and *Medicago*, disruption of secondary cell wall synthesis can result in the prevention of anther dehiscence [26, 42, 43]. Reverse genetic characterization of *MTIRX1* provides proof of concept that the transcriptome data may serve as a valuable resource for further secondary cell wall gene discovery in *M. truncatula*.

## Discussion

The inflorescence stem of the herbaceous model plant *Arabidopsis* forms vascular xylem and undergoes secondary growth, and has therefore been used to study secondary cell wall development [44, 45]. The advantages of studying *Arabidopsis* include its known genome, easy transformation and available mutant resources for functional studies. For alfalfa and other legume species, *M. truncatula* may be a better model system due to the high homology of their genomics sequences. In some cases, genes identified from *M. truncatula* can be directly utilized in alfalfa [13]. A high quality genome sequence of *M. truncatula* is available [8]. A gene expression atlas with a global view of gene expression in all major organs, and a large collection of *Tnt1* retrotransposon insertional mutant lines available for gene functional analysis [27, 46, 47] make *Medicago* an excellent model for functional genomics of cell wall development in legumes.

The cluster analysis of lignin and cellulose biosynthetic genes indicates that most of the genes are correlated with secondary cell wall development as supported by characterization of the cellulose synthase gene *MtIRX1*. However, some of the putative lignin and cellulose synthase genes were categorized in cluster 1, which showed opposite expression patterns compared to genes in secondary cell wall development (Fig. 4a and b). The functions of these gene are still unclear. One possibility is that these genes may function in cell wall development in certain conditions other than normal growth conditions. This is supported by functional analysis of the cinnamoyl CoA reductases (CCR) genes in *Medicago* [48]. Mt*CCR1*, identified in this previous study as the primary *CCR* gene in the monolignol biosynthetic pathway, is categorized in cluster 10. Mutation of *MtCCR1* resulted in drastic inhibition of plant growth. In contrast *MtCCR2* is in cluster 1, and its mutation has no effect on plant growth [48].

The high-throughput real time qRT-PCR analysis identified 64 transcription factor genes that significantly up-regulated along the stem maturation gradient. Among these, twenty were already detected in the microarray experiment. The overlapping transcription factors between Genechip and qRT-PCR is low mainly because these two methods have obvious difference in sensitivity. We found that eighteen TFs changed less than 2-fold in the microarray assay, but changed over 5 times in the qRT-PCR analysis, indicating that qRT-PCR is much more sensitive than the Genechip assay. In addition, the annotation of *M. truncatula* genome sequence is still far from complete. Even though the *M. truncatula* Genechip have 1394 probe sets corresponding to transcription factor genes, we found 26 TFs significantly differentially expressed in qRT-PCR analysis, but were not present on the Genechip. The GeneChip approach heavily depends on chip design. For example, the current *M. Truncatula* chip contains only about 70-75 % of the genome contents [27]. In addition, the Genechip can’t differentiate closely related genes, which can be addressed by the complementary real time qRT-PCR approach.

## Conclusions

In this study, we found that over 11,000 genes are differentially expressed along the maturation axis of *Medicago* primary stems. It was previously estimated that about 15 % of the genes in *Arabidopsis* may be involved in cell wall synthesis, remodeling, or turnover [49]. It is likely that many of the differentially expressed genes play important roles in secondary cell wall development. However, stem development along the maturation axis involves many other developmental processes, including the maturation of ground tissues. The transcriptome expression pattern should also reflect the development of these tissues. To identify genes with specific function in secondary cell wall development, we have here analyzed the transcriptome data with further consideration of expression specificity. Genes with enhanced expression in secondary cell wall bearing tissues, or co-expressed with wall biosynthetic genes were selected for further characterization. These analyses should help to reduce the false discovery rate. Bioinformatics analysis together with reverse genetics studies are especially helpful to pinpoint the genes involved in secondary cell wall formation [50], and the present confirmation of the function of *MtIRX1* highlights the potential of the present dataset for further cell wall gene discovery.

## Methods

### Plant materials and growth conditions

Wild type *M. truncatula* plants and *Nicotiana tabacum* (tobacco) *Tnt1* retrotransposon tagged Medicago mutants [47] were grown in the glasshouse, and used for collecting tissues for molecular and chemical characterization. Plants were grown at 24 °C day/20 °C night, with a 16-h day/8-h night photoperiod (150 μmol m^-2^ sec^-1^) and 70–80 % relative humidity.

### Microarray analysis

Stems of 7 weeks old *M. truncatula* plants (totally 10-11 internodes) were used for sampling. Internodes 2, 3, 5, 7 and 9 counting from the plant tip were collected. Total RNAs were extracted using tri-reagent according to the manufacturer’s protocol (Invitrogen). RNA was cleaned and concentrated using the RNeasy MinElute Cleanup kit (Qiagen, http://www.qiagen.com). Ten micrograms of purified RNA were used for microarray analysis. The microarray experiment included five internodes with three biological replicates, used 15 Affymetrix Genechips in total. Probe labeling, hybridization and scanning were conducted according to the manufacturer’s instructions (Affymetrix, http://www.affymetrix.com). The normalization of data was achieved by the robust multi-chip average (RMA) procedure [51]. The presence/absence call for each probe set was obtained from dCHIP [52]. Genes significantly differentially expressed between controls and mutants were selected using Associative Analysis [31]. The type-I family-wise error rate was reduced using a Bonferroni corrected *P*-value threshold of 0.05/n, where n represents the number of genes present on the chip. The false discovery rate was monitored and controlled by Q-value (false discovery rate), calculated using EDGE (extraction of differential gene expression; http://www.bioconductor.org/packages/release/bioc/html/edge.html) [53, 54]. Hierarchical clustering analysis was conducted with Spotfire DecisionSite 8.1 (Spotfire Inc., http://spotfire.tibco.com/). For clustering analysis, data from different internode were expressed as relative to the level of IN2 just prior constructing clusters using the Pearson correlation coefficient.

### Real-time PCR

Real-time PCR analysis of 1045 transcription factor genes were carried out at the Genomics/Microarray core facility at the Samuel Roberts Noble Foundation. Quantitative real-time PCR (qRT-PCR) and the calculation of relative expression were performed as described previously [35]. In brief, cDNA samples prepared from the aforementioned five internodes were used for real time RT-PCR with technical duplicates. The 10 μl reaction included 2 μl of primers (0.5 μM of each primer), 5 μl of Power Sybr (Applied Biosystems, http://www.appliedbiosystems.com/absite/us/en/home.html), 2 μl 1:20 diluted cDNA from the reverse transcription step, and 1 μl of water. Real-time RT-PCR data were analyzed using SDS 2.2.1 (Applied Biosystems). The community high-throughput qRT-PCR uses a 384-well plate format with eight reference genes included on each plate. Information of primer sequences, accession numbers and stability test were described previously [37]. Transcript levels were determined by relative quantification using the Medicago Ubiquitin gene (TC102473, primers: UbiFw, GCAGATAGACACGCTGGGA; UbiRe, AACTCTTGGGCAGGCAATAA) as a reference. Amplification efficiency (E) was determined from three biological replicates of each of the five internode samples using LinRegPCR [55]. The relative expression of all internodes to internode 2 was calculated using the mean of three biological replicates for each organ. A TF gene was considered increased or decreased only if transcript levels for that gene were changed 5-fold or more than those of internode 2.

### Gene co-expression network analysis

Expression data were collected from the Medicago Gene Expression Atlas [27]. Data for leaf, petiole, stem, shoot (split-root exp) sufficient N_2_, flower, pod, root and split root (nodulating) sufficient N_2_ and the present stem experiment (37 chips in total) were used. Only the probe sets with at least two-fold change in comparing the maximal value to the minimal value in stem experiments were selected. We also removed the probe sets “AFFX”, “Sme” and “RPTR”. Thus, 12,576 probe sets remained. To generate a gene co-expression gene network for those selected probe sets, we used our in-house R script to calculate the Pearson correlation coefficient (PCC) values for all 79,071,600 (=12576 × (12576-1)/2) probe set pairs in the above dataset. Two probe sets (genes) are considered linked if their observed correlation level (|PCC|) exceeds a significant threshold value. To estimate this threshold, we permutated the expression values for each probe set independently to generate a random dataset following an approach proposed by Carter et al. [56]. We calculated PCCs for all probe set pairs in this random dataset, and obtained the distribution of PCC frequency from -1 to 1 at a 0.1 interval. From this distribution, we observed that no probe set pair that can reach |PCC| > 0.8 in the random dataset (Additional file 1: Figure S2), indicating that 0.8 is a statistical significant level to determine whether there is a connection between each probe set pairs in the network. As a result, a co-expression gene network is produced for further analysis. The TFs that are connected with lignin, cellulose and hemicellulose biosynthetic genes are presented as three sub-networks in the results (Fig. 6). The visualization of gene networks is implemented by an open source software Cytoscape [57].

### Cell imaging and histochemical staining

To characterize the *Tnt1* insertional mutants, the sixth internodes counting from the top of the plant were collected from plants grown in the glasshouse and immediately frozen in liquid nitrogen. Cross-sections (100 μm) of the sixth internodes were cut with a Leica CM 1850 cryostat (Leica Microsystems Inc., Buffalo Grove, IL, USA) at-20 °C and prepared for microscopy as previously described [58]. Phloroglucinol-HCl staining was carried out as previously described [25]. Photographs were taken with a Nikon Micophot-FX system (http://www.nikon.com) with a Nikon DXM 1200 color camera with consistent settings.

### Availability of supporting data

The Microarray data sets supporting the results of this article is available at ArrayExpress with a accession number: E-MTAB-3909

## Additional file

Additional file 1: Supplemental Figure 1. Expression pattern analysis of cluster 10 transcription factor genes. Supplemental Figure 2. The PCC distributions in the real and random datasets. Supplemental Table 1. Distribution of differentially expressed genes in the 10 clusters. Supplemental Table 2. Probe sets and corresponding names of lignin biosynthetic genes. Supplemental Table 3. Transcription factor genes upregulated during stem maturation.
