# Supplementary material for: Transcriptome analysis of secondary cell wall development in Medicago truncatula
Source: BMC Genomics. 2016 Jan 5;17:23. doi: 10.1186/s12864-015-2330-6 (PMC4700669; doi:10.1186/s12864-015-2330-6)
Supplement: Additional file 1: — Supplemental Figure 1. Expression pattern analysis of cluster 10 transcription factor genes. Supplemental Figure 2. The PCC distributions in the real and random datasets. Supplemental Table 1. Distribution of differentially expressed genes in the 10 clusters. Supplemental Table 2. Probe sets and corresponding names of lignin biosynthetic genes. Supplemental Table 3. Transcription factor genes upregulated during stem maturation. [file 12864_2015_2330_MOESM1_ESM.pdf]

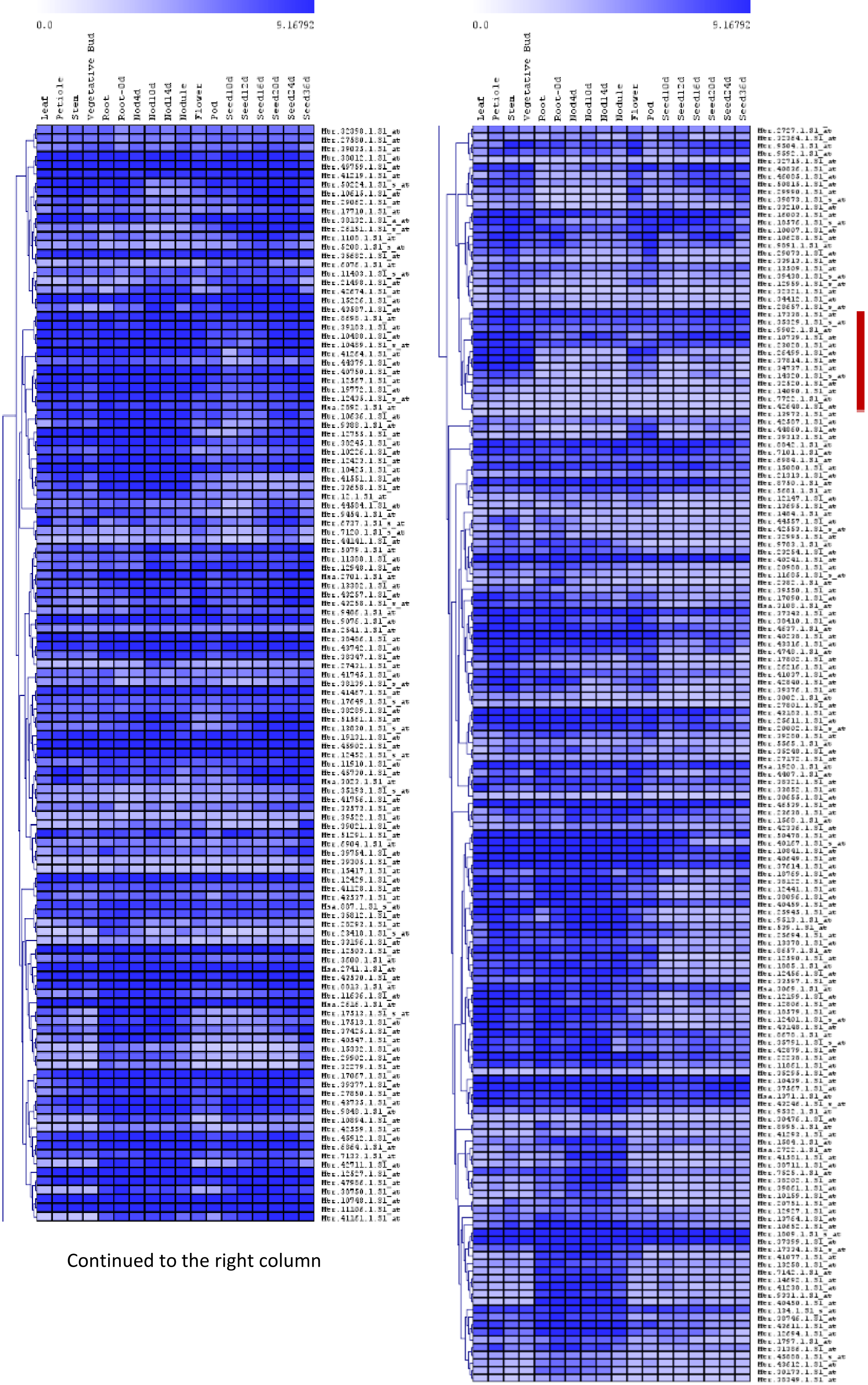

Supplemental Figure 1. Expression pattern analysis of cluster 10 transcription factor genes. Expression of all selected 279 transcription factors was analyzed based on expression patterns in different tissues. Red bar highlights the clade with genes highly expressed in secondary wall bearing tissues.

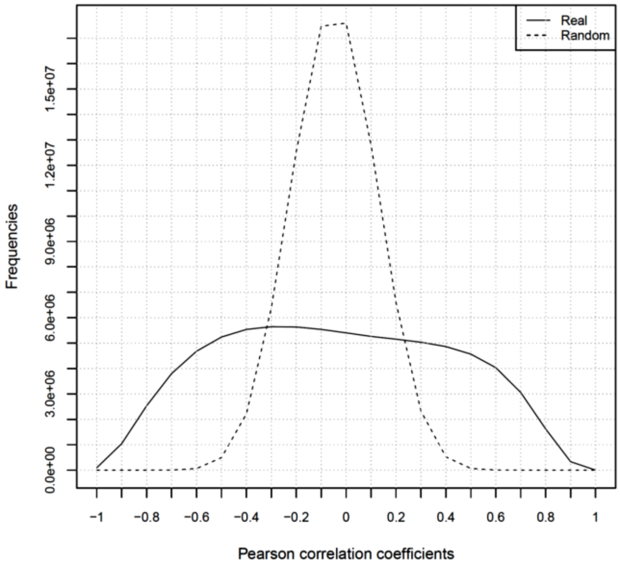

Supplemental Figure 2. The PCC distributions in the real and random datasets.

Supplemental Table 1. Distribution of differentially expressed genes in the 10 clusters.

Cluster	Genes	Gene number
Cluster 1	1-5068	5068
Cluster 2	5069-5093	25
Cluster 3	5094-5173	80
Cluster 4	5174-5278	105
Cluster 5	5279-5393	115
Cluster 6	5394-5636	243
Cluster 7	5637-5646	10
Cluster 8	5647-5930	284
Cluster 9	5931-6160	230
Cluster 10	6161-11380	5220

Supplemental Table 2. Probe sets and corresponding names of lignin biosynthetic genes.

Probe sets	gene name
Mtr.27096.1.S1_at	CAD1
Mtr.15494.1.S1_at	CAD2
Mtr.8589.1.S1_at	CAD3
Mtr.40241.1.S1_at	CCR1
Mtr.37453.1.S1_at	CCR2
Mtr.5789.1.S1_s_at	CCR3
Mtr.40238.1.S1_at	HCT
Mtr.40166.1.S1_s_at	PAL1
Mtr.50478.1.S1_at	PAL2
Mtr.51909.1.S1_at	PAL3
Mtr.40167.1.S1_s_at	PAL4
Mtr.40168.1.S1_at	PAL5
Mtr.10404.1.S1_at	PAL6
Mtr.13904.1.S1_at	4CL1
Mtr.42337.1.S1_at	4CL2
Mtr.2048.1.S1_at	4CL3
Mtr.25611.1.S1_at	4CL4
Mtr.41031.1.S1_at	4CL5
Mtr.9793.1.S1_at	4CL6
Mtr.44778.1.S1_at	4CL7
Mtr.45628.1.S1_s_at	4CL8
Mtr.12556.1.S1_at	4CL9
Mtr.43335.1.S1_at	4CL10
Mtr.10628.1.S1_at	F5H1
Mtr.42553.1.S1_s_at	F5H2
Mtr.43183.1.S1_at	C3'H
Mtr.5380.1.S1_s_at	COMT1
Mtr.2631.1.S1_at	COMT2
Mtr.33866.1.S1_at	CCOAOMT1
Mtr.4850.1.S1_at	CCOAOMT2
Mtr.31539.1.S1_at	CCOAOMT3
Mtr.40942.1.S1_at	CCOAOMT4

Supplemental Table 3. Transcription factor genes upregulated during stem maturation.

Gene Identifier	Internode 2	Internode 3	Internode 5	ternode 7	ternode 9
759.m00018	1	3.63345811	189.0775284	59.036592	25.513702
816.m00024	1	79.6886871	115.8955838	30.39231	27.084158
IMGAG|67.m00243	1	6.43708517	112.4012858	303.60123	529.45221
1039.m00012	1	4.67954936	84.19203715	243.05015	655.68848
919.m00002	1	8.96058732	76.24061311	92.397773	93.665549
1552.m00024	1	1.16418937	70.37779419	71.671223	70.762852
784.m00022	1	2.22011466	64.96715652	196.95909	284.25787
1648.m00026	1	45.8701547	63.84414708	65.017498	63.139623
1400.m00006	1	-	58.25908597	59.329793	40.701912
956.m00007	1	4.75128524	54.33709953	113.44689	118.14878
1001.m00016	1	5.08546495	51.70592224	70.928851	109.79609
1475.m00011	1	4.42438767	31.15647814	31.729083	45.917896
1044.m00008	1	4.43887985	26.08805568	25.23761	32.078271
1287.m00026	1	2.41774545	25.83601418	26.614463	22.26833
72.m00191	1	14.4249749	23.9439933	28.210625	20.798893
1183.m00022	1	10.7650479	20.64116774	139.75348	113.94369
1261.m00007	1	1.17969365	19.41797759	55.106401	104.16736
749.m00018	1	2.62577183	19.00500407	8.5372556	27.324216
1374.m00048	1	1.47981277	18.47347228	28.970722	37.76196
1047.m00031	1	1.28866374	16.87895388	19.750541	23.316563
119.m00199	1	1.24645339	16.85930007	11.79638	11.763622
812.m00002	1	6.79012493	14.81215425	16.545108	12.08619
1235.m00016	1	7.07459411	14.63032264	9.8345092	8.7846613
1230.m00008	1	7.08189971	14.55519932	37.779085	28.559774
1434.m00003	1	5.55394109	14.50908519	14.775739	9.1780328
1677.m00002	1	8.6898935	13.21318217	13.456019	12.234704
1194.m00013	1	1.83015422	12.12049395	18.126909	21.237903
773.m00019	1	0.47637024	12.10811336	9.2260828	10.734591
1064.m00002	1	3.16480456	11.04030543	10.146225	13.751037
1376.m00003	1	2.90913461	10.96450655	8.6923504	7.4812777
1042.m00016	1	4.39277692	10.34381191	8.972535	8.1205021
980.m00009	1	1.78175995	10.25772254	44.40529	26.014346
995.m00006	1	1.85474743	9.906599235	10.088666	3.104871
743.m00017	1	9.60796153	9.725114163	9.9038459	7.1971703
1313.m00037	1	2.51965438	9.305416631	8.1717825	7.0313796
1254.m00011	1	17.8317741	9.018490265	9.1842354	6.0754716
766.m00018	1	1.87118574	8.339858639	8.4931317	14.135096
1254.m00006	1	8.2212015	8.282707381	8.43493	6.2252957
1266.m00032	1	3.10846272	8.213452972	1.6775735	3.5790541
920.m00017	1	5.72124053	8.168561426	6.9112312	7.8245137
1411.m00015	1	0.61902354	8.033002419	8.1806359	15.043453
822.m00019	1	1.20737805	8.012818552	8.1600811	15.014617
1567.m00020	1	-	7.738856222	7.8810838	4.0400358
1224.m00021	1	0.80738305	7.585769784	5.4107386	2.7175285
1347.m00026	1	2.16444069	7.536807194	4.639206	2.3443542
1425.m00008	1	2.69413233	7.493117076	7.6308284	14.026759
736.m00014	1	7.35200386	7.441085278	7.7857829	5.7210675
739.m00012	1	0.37525541	7.380855958	8.8694339	14.817364
1015.m00015	1	1.8988633	7.170599088	9.6827562	7.7827185
905.m00015	1	2.14178221	6.448987615	11.285783	12.973148
930.m00011	1	2.6043995	6.322752745	8.7282489	9.8942844
767.m00007	1	0.95645439	6.212044426	0.6483232	8.9671318
135.m00197	1	2.60315015	5.985657401	6.7237136	8.4622988
1276.m00011	1	2.36717091	5.667142001	5.7712948	3.0678256
747.m00011	1	0.22711225	5.385970359	6.4260414	5.8869712
1140.m00014	1	1.64603576	5.306243344	6.6518426	6.3839346
988.m00017	1	1.28453695	4.783488647	5.9722148	6.0681684
1147.m00014	1	2.07984479	4.55827461	6.7837162	6.989861
1316.m00009	1	1.3256682	4.519416844	39.438006	46.142964
918.m00003	1	1.14057427	4.366544499	6.7593575	8.2168112
1084.m00021	1	2.11100153	4.068318923	5.7671053	4.2979086
1705.m00010	1	0.95471663	4.000708833	4.0742353	8.3415816
972.m00008	1	2.84012058	3.038069302	7.5051991	8.6395952
1047.m00030	1	0.95835404	2.612938341	14.248976	19.802758
